# Acknowledgment to the Reviewers of *Journal of Intelligence* in 2022

**DOI:** 10.3390/jintelligence11010014

**Published:** 2023-01-13

**Authors:** Journal of Intelligence Editorial Office

**Affiliations:** MDPI AG, St. Alban-Anlage 66, 4052 Basel, Switzerland

High-quality academic publishing is built on rigorous peer review. The *Journal of Intelligence* was able to uphold its high standards for published papers due to the outstanding efforts of our reviewers. Thanks to the efforts of our reviewers in 2022, the median time to first decision was 25 days and the median time to publication was 64 days. Regardless of whether the articles they examined were ultimately published, the editors would like to express their appreciation and thank the following reviewers for the time and dedication that they have shown *Journal of Intelligence*:A. Halim M. SiKaarin J. AnsteyA. Jordan WrightKani UlgerAakash ChowkaseKarina AdboAdam ChuderskiKarl SchweizerÅge DisethKarthik H. ShankarAgnieszka KonysKatarzyna BobrowiczAgnieszka WojtowiczKatherine L. McNeely-WhiteAhmad Zamri KhairaniKathryn McCarthyAhmed M. Abdulla AlabbasiKees Jan KanAkhtar AkhtarKeith SawyerAlan S. KaufmanKenneth GilhoolyAlberto Rubén OsellaKevin Paul MadoreAlejandro Valencia-AriasKevin SchilbrackAlenka KavčičKim C. BrimhallAlexander BeaujeanKim Van BroekhovenAlexandra LenhardKinga StecułaAlexandre AubryKit S. DoubleAlexandre MaroisKristin JankeAlina IvanovaKristin StoefflerAljoscha NeubauerKristof KovacsAllan FowlerKrisztian JozsaAlmudena Alonso-FerreiroKwan Eu LeongAmal KhalifaKyoung Shin ParkAmber WitherbyLaTasha R. HoldenAna María Botella NicolásLaura Dörrenbächer-UlrichAna Paula AiresLaura JandaAna Rodriguez-GrobaLaura MalininAna VivasLaurentiu TiruAndrás BenedekLawrence Jun ZhangAndre BeauducelLeehu ZysbergAndré KretzschmarLetchmi Devi PonnusamyAndrea GrausLinda JarvinAndreas DemetriouLindsay D. OliverAndreas DengelLindsay Ellis LeeAndreas ZehetnerLisa K. SonAndreja ŠpernjakLisa WagnerAndrew LarnerLiviu-Adrian CotfasAnita Pásztor-KovácsLorenzo FiorineschiAnja KühnelLouis HickmanAnna Maria DådermanLuísa SoaresAnna ZiomkiewiczŁukasz SmagaAnna-Lena SchubertLuke D. SmillieAnne M. RobertsLyubka AleksievaAnne TraynorM. Angeles QuirogaAntigoni ParmaxiM. Danish ShakeelAntonia Pades JimenezMagda Di RenzoAntonia SzymanskiMaja WojciechowskaAntonio QuesadaManuel PeralboAntonio Vicente Rodríguez FuentesMarco LauriolaAriel BelasenMarco SantorumArto K. AhonenMarcos SantosAshley L. MillerMaría Cristina Martínez-FernándezAthanasios S. DrigasMaria Cristina Triguero Veloz TeixeiraAthanassios AndroutsosMaría Luisa Zagalaz SánchezAttila PásztorMaria PlatsidouAzlina Binti Mohd KosninMaria VenderBaoguo ShiMaría-Leticia Meseguer-SantamaríaBarbara HanfstinglMariana V.C. CoutinhoBeate EichmannMarianna M. WalkerBen WeidmannMarina FioriBenjamin LovettMario DalmasoBernhard HommelMarisha HumphriesBeth Helen RichardsonMarita CaninaBeth Mozolic-StauntonMark BrayBirgitta Dresp-LangleyMarkus ChristinerBo ZhangMarta Sainz-GómezBoris ForthmannMartin HechtBrandon KohMartin MičiakBrian M. FrankMateus JoffilyBridget SmeekensMatevž PesekBrooke MacnamaraMathupayas ThongmakBruno FaconMatt BrownCallie LittleMatteo ChiappediCameron O’Neill ByerleyMatthew C. MakelCarlos SalaveraMatthew J. EulerCarmen Flores-MendozaMatthew ReynoldsChao HuMatthew RhodesChen ChengMatthias ZieglerChet RobieMattis GeigerChiara FinocchiaroMaud BesanconChin Yen HanMaura M.E. PilottiChristian Campos-JaraMauricio Castillo VergaraChristian Maximilian ThurnMd. Sayeed Al-ZamanChristoph StrauchMeesha WarmingtonChristopher DraheimMehdi GhahremaniChristopher HopwoodMelanie S. MeyerChuanhua GuMelissa HawthorneClark A. McKownMichael S. MatthewsClaudia Von BastianMichał BronikowskiClaudio Cubillos FigueroaMichał CzubenkoColin CooperMichelle L. RiversColleen M. SeifertMiguel Alcázar-CórcolesCorentin GonthierMihaela MoscaluCorey ClelandMikhail V. NoskovCristian CechinelMilagros Fernández MolinaCristian Tejedor-GarcíaMing LiCsíkos CsabaMohamed El HajjiDamian BirneyMoritz BreitDan SongMoshe ZeidnerDandan TongNaemi BrandtDaniela TraficanteNancie Im-BolterDavid GoretzkoNapp ClotildeDavid I. AndersonNaser AqababaiDavid MoleroNicolette WaschlDavid MunezNieves Fátima Oropesa RuizDavid PreissNikša AlfirevićDavid R. PillowNils MyszkowskiDavid ReillyNina KrügerDean Keith SimontonNing HaoDeanna FriesenNora MurphyDeborah L. ButlerNurit Paz-BaruchDeia GanayimmNuttida RungratsameetaweemanaDejan BudimirovicÖkördi RékaDenis BratkoOphelie Allyssa DesmetDiego Sánchez-GonzálezOriol Borrás-GenéDobrinka KuzmanovicÓscar Gavín ChocanoDobromir RahnevPalmira PečiuliauskienėDorothy SiskPaloma Barjola ValeroEda KöksalPanaoura AretiEdgardo EtchezaharPascale VoelkerEimear McGlincheyPatrick PerretEkaterina OrelPatrizia PiottiEleanor R. PalserPaul De BoeckElena ZaretskyPaul RouxEleni BontiPaula Cifuentes-FérezEleni MitseaPaula VianaElif Tekin IftarPavel SmolkaElisavet ChrysochoouPedro SanchezElizabeth A. CanningPedro VideiraEloy López MenesesPeerayuth CharoensukmongkolElsbeth SternPei-Ying ChenÉmilie DujardinPeter F. DelaneyEmily A. WilloughbyPéter KörtesiEmily FarranPeter OrganisciakEric KloppPeter P. UjmaEster NavarroPhilip FineEufrasio Pérez NavíoPhilipp SonnleitnerEvgeniya KorotaevaPhillip L. AckermanF. Richard FerrarPing ZouF. Richard OlenchakPoh Kiat NgFabian ZehnerPrzemysław Robert NowakowskiFa-Chung ChiuQin ZhaoFei GuoQing WangFernando Manuel Lourenço MartinsQiwei HeFlorian SchmitzQunlin ChenFrancesca BorgonoviRadek KrpecFrancesco AmentaRadwa KhalilFrancisco RiveroRakefet AckermanFrank GoldhammerRandall S. DaviesGary N. MarksRea LaviGeorgi GeorgievRebecca MarroneGeorgia PapantoniouRenato BorgattiGerolf RennerRex JungGidon FrischkornRichard KumiGill WatersRichard MayerGino CasaleRobert A. BjorkGiovanni E. CorazzaRobert GaschlerGiusi Antonia TotoRobert J. SternbergGizem HülürRoberto ColomGlenn Ole HellekjærRogelio Puente-DiazGonny SchellingsRomina Cecilia ElisondoGordon B. SchmidtRuilin WuGunter KreutzRupesh Kumar ChikaraGuy TraininSabine PirchioGyöngyvér MolnárSamy GhoniemyHaijun DuanSandra KaplanHans LuytenSanne F. E. RoversHao WuSantos Orejudo HernándezHao XuSareh KaramiHaobo ZhangSeongjin AhnHarrison KellShahaf S. ShperbergHasan BedirShengjie LinHeather KemptonShifang TangHeng LuoShira ElqayamHeribert Harald FreudenthalerSilvana MarevaHung-Hsiang WangSimon HandleyIda Rosnita IsmailSimona Catalina StefanIgor BajšanskiSri TatminingsihIngo KollarStamatios PapadakisInsa FeinkohlStamatis KarlosIrene Ferrando PalomaresStefan TrocheIrene P. CarvalhoStefanie VanbecelaereIrini MavrouStefano FerilliIryna AshbyStephanie MillerIuliia PavlovaStephen M. FioreIvailo PartchevSteve EntrichIvana BianchiSteven Lloyd WilsonIvana KovačevićSteven PanJ. Gallardo-PérezSteven ShawJacqueline M. CaemmererSteven StemlerJakob PietschnigSucheta KolekarJames J. LeeSusana PintoJames KaufmanSusana SilvaJan RummelSusanne PodwornyJana JungjohannTaffeta WoodJana UherTarquino Sánchez-AlmeidaJanet Frances RafnerTatyana Vasilievna KornilovaJanet MetcalfeTengfei WangJaroslav BeránekTetyana PimonenkoJason R. ParkinTheodore ChristJavier CorbalánThierry LecerfJeff CranmoreThomas J. WardJeffery BradenThomas R. CoyleJeffrey HerrmannThomas Rhys EvansJens F. BeckmannThomas RockstuhlJeroen LavrijsenTodd LubartJessica HoffmannTomer SagiJessica M. NamkungTommaso FeracoJessica McKenzieTomoe KanayaJi HanTuuli TurjaJia UddinÜzeyir OgurluJian LiValerie StoreyJiangjie ChenVanessa Arán-FilippettiJiumin YangVas TarasJoanna SamulVeronica YanJoaquin TavernerVeronika StoffaJochanan VeerbeekVincent AlfonsoJoel ParthemoreVincenza CofiniJohannes RodriguesViola OldratiJohannes SchultVittorio FineschiJohn D. MayerVsevolod ScherrerJohn ProtzkoW. Joel SchneiderJolanta MackowiczWang-Kin Oscar ChiuJolanta SzempruchWei WangJonas LangWenjing YangJonathan PluckerWenke MöhringJonathan WaiWieslawa LimontJongbeom LimWilhelmina Van DijkJosé Antonio GarzónWilliam J. BeckerJosé Manuel Sáez LópezXiaobo XuJosé Paulo CravinoXiaofei WuJoseph AllenXiaoqian YuJoshua J. PrasadXiaowen JiJozsef KatonaXinwen BaiJuan BetancurXuesong (Andy) GaoJuan González-MartínezXuezhu RenJuan M. Trujillo-TorresYadan LiJudit García MartínYuliya ShtaltovnaJulie A. TeichroebZhihui CaiJulio AcostaZhijin ZhouJumadil SaputraZsuzsanna KöviJunlong Luo



